# *Astragalus* Oral Solution Ameliorates Allergic Asthma in Children by Regulating Relative Contents of CD4^+^CD25^high^CD127^low^ Treg Cells

**DOI:** 10.3389/fped.2018.00255

**Published:** 2018-09-20

**Authors:** Wei Wang, Wei Jing, Qingbin Liu

**Affiliations:** Department of Pediatric, Affiliated Hospital of Changchun University of Chinese Medicine, Changchun, China

**Keywords:** *Astragalus* oral solution, Astragaloside A, allergic asthma, CD4^+^CD25 ^high^CD127^low^ Treg cells, children

## Abstract

**Objective:** To explore the effects of *Astragalus* oral solution (AOS) on allergic asthma in children by investigating relative contents of CD4^+^CD25^high^CD127^low^ Treg cells.

**Methods:** The contents of Astragaloside A in AOS were detected by using HPLC. Eighty children with allergic asthma were recruited from February 2016 to June 2017, and randomly assigned into the control group (received placebo, 0.1% quinine chloride in deionized water, daily) and the AOS group (received 10 mL AOS daily). After 6-month treatment, therapeutic results were compared between the two groups. Serum levels of IL-10 and TGF-beta, Th1 cytokines (IL-2 and IFN-γ), and Th2 cytokines (IL-4 and IL-6) were measured by using ELISA kits. Relative contents of CD4^+^CD25^high^CD127^low^ Treg cells were determined by using flow cytometry.

**Results:** Astragaloside A was the main ingredient of AOS with 0.216 ± 0.027 mg/mL from six-batch samples. After 6-month therapy, the AOS group showed improved forced expiratory volume in 1 s (FEV1) and the Pediatric Asthma Quality of Life Questionnaire (PAQLQ) scores compared with the control group (*P* < 0.05). Serum level of IL-10 was higher and the levels of TGF-beta, Th1 cytokines (IL-2 and IFN-γ), and Th2 cytokines (IL-4 and IL-6) were lower in the AOS group than in the control group (*P* < 0.05). AOS treatment increased the percentage of gated CD4^+^ T cells, CD4^+^CD25^+^ T cells, CD4^+^CD25^high^ Treg cells, CD4^+^CD25^+^FoxP3^+^ Treg cells and CD4^+^CD25^high^CD127^low^ Treg cells when compared with the control group (*P* < 0.05).

**Conclusions:** Astragaloside A was the main component of AOS, and AOS ameliorated allergic asthma in children by regulating relative contents of CD4^+^CD25^high^CD127^low^ Treg cells.

## Background

Asthma is a common inflammatory disorder of the lungs and is often characterized by reversible airflow obstruction and bronchospasm. Allergic asthma is a main threat to public health as it perturbs children's respiratory system. More than 300 million people worldwide have allergic asthma (1, 2). According to the data from the World Health Organization (WHO), asthma affects most children, and the number is expected to increase to 400 million in 2025 (3). With the development of society and industrialization, the incidence and mortality of allergic asthma in children have been increasing. Approximately 30–90% of children with allergic asthma have allergic rhinitis (AR) (4). AR is one of the related factors in the pathogenesis of allergic asthma, which leads to a direct result of asthma onset (5). With their similarities in etiology, location, pathogenesis, and pathophysiology, both AR and allergy asthma should be treated together (6). Seasonal perennial allergens, such as grass, trees, pollen, house dust (HD), mold, and animal fur, can often cause allergic asthma in children (7, 8). In allergic patients, respiratory allergies often lead to systemic allergies. In nasal allergic inflammation, Th1, Th2, and Th17 cells migrate into the bone marrow, stimulating the production of inflammatory cells, mast cells, IL-5, IL-13, IL-17, IL-22, and IL-33 (9). Inflammatory cells and cytokines enter the nasal mucosa and lungs, triggering respiratory airway inflammation and causing allergic symptoms (10). At the same time, they can also increase the expression of adhesion molecules in the nasal and bronchial mucosa, thereby aggravating respiratory allergies (11).

Studies have shown that eosinophils and mast cells play important roles in the pathogenesis of allergic asthma (12). Th2-like cytokines are mainly secreted by T cells and regulate asthma airway inflammation. T cells and interleukins have been found to play an important role in the progression of allergic asthma (13).

The regulatory T cell (Treg) is a subpopulation of T cells that regulate the immune system, maintain tolerance to self-antigens, and prevent autoimmune disorders. An earlier report found decreased Treg cells in patients with asthma, and there was a significant correlation between change in airway Tregs cells and asthma. Improving Treg cells may be a novel strategy in the prevention of asthma and other allergic disorders (14). CD4^+^CD25^+^ Treg cells are a unique population of Treg cells that can independently modulate adaptive and innate autoimmune responses (15). Foxp3 is a cell marker of CD4^+^CD25^+^ Treg cells and is closely related to the differentiation and function of regulatory Treg cells (16, 17), which can regulate the immune responses. In the pathogenesis of asthma, Treg cells secrete a variety of cytokines that can suppress the proliferation of T cells and the synthesis of IgE through the transmission of inflammatory cells, such as IL-10 and TGF-β. In different stages of allergic asthma, the analysis of the effects of cytokines and chemokines becomes very important for asthma prevention and treatment.

*Astragalus*, a large genus of herbs, belongs to the legume family Fabaceae and has potent immune boosting and health-promoting properties (18, 19). *Astragalus membranaceus* (AM) has been widely used for thousands of years in China to treat asthma. Animal model tests showed that the extracts of AM can increase the levels of CD4^+^ CD25^high^ Treg cells and Foxp3 mRNA expression in an asthmatic animal model (20). *Astragalus* was further found to prevent the recurrence of asthma by modulating Th1/Th2 cytokines in asthmatic children (21). *Astragalus* oral solution (AOS) is made by the Affiliated Nanjing Hospital of Nanjing Medical University, and one of active ingredients is Astragaloside A, which regulates immune responses. Despite the high efficacy of AOS, little is known about the changes of IL-10, TGF-β, Th1, and Th2 cytokines, and CD4^+^CD25^high^CD127^low^ Treg cells in the children with allergic asthma. Children with allergic asthma were recruited at our hospital from 2016 to 2017, and treated by using AOS. Related indicators and immunological changes were examined to evaluate the value of AOS treatment for the children with allergic asthma.

## Materials and methods

### Materials

Astragaloside A was purchased from China Pharmaceutical and Biological Laboratory (lot number 11078-200703, Beijing, China). AOS was purchased from Nanjing Hospital of Traditional Chinese Medicine (patches numbers: 100234,100406, 100609, 100815, 100987, and 101009; Nanjing, China). AOS was prepared as follows: AM slices were dried to a constant weight, 450 g. After soaking in 5 liters of distilled water overnight, the main ingredients of AM were extracted by using the ultrasonic constant temperature ultrasonic extractor (Cat. No., Scientz-5TQL4, Ningbo Scientz Biotech., Ningbo, China) twice. The first extract was obtained at 30 min (1000 W), and the second tract at 45 min and 50°C, and the extracts were concentrated to 450 mL. The extracts were further centrifuged at 12,000 × *g* for 30 min, and supernatants were filtered through a 10 kDa membrane (Millipore, Bedford, MA, USA).

### Chromatographic conditions

The HPLC system consisted of a Kromasil C18 column (4.6 × 200 mm, id 5 μm) with a guard column (10 × 4.6 mm, id 5 μm) (Waters Chromatography Division of Millipore Corp., Milford, MA, USA), a Waters 515 HPLC Pump, a full-wavelength UV detector, a Waters 717 plus autosampler injector and an Empower workstation. The mobile phase was composed of acetonitrile-water (35:65, v/v), the determination wavelength was 203 nm, and the column temperature was 30°C. All reagents were of chromatographic grade. To achieve the baseline separation and accurate quantitative analysis, the theoretical plate number of Astragaloside A was not less than 3,000.

### Measurement of astragaloside A in AOS

One-milliliter of AOS was added to a 10 mL volumetric flask and extracted twice with petroleum. The petroleum ether extract was discarded, and the lower solution was extracted four times with saturated n-butanol (20, 15, 15, and 10 mL). The combined n-butanol solution was washed with 5% sodium bicarbonate solution twice, each for 30 mL. The washings were discarded and n-butanol solution was collected, and evaporated to dry. The residue was dissolved in 10-mL ethanol. Approximately 0.05 g Astragaloside A standard was added to a 25 mL volumetric flask, and ethanol was added to achieve 1.967 mg/mL Astragaloside A as a reference. A series of the stock solution (0.2, 0.4, 0.6, 0.8, and 1.0 mL) was added to 10 mL volumetric flask, and ethanol was added to achieve a series of Astragaloside A standards with different concentrations (0.0393, 0.0786, 0.118, 0.157, 0.196 mg/mL). Ten microliter of solution was injected into HPLC and the peak area of Astragaloside A was determined. The measurements were repeated twice. The peak area was used as the ordinate, Astragaloside A concentration was used as abscissa, and the standard curve was plotted. The regression equation was y = 1.245x + 7.526C, *r* = 0.9996. The standard was repeated for five times with RSD 0.7% to ensure the method was accurate. AOS was measured by using the same situation and the results showed perfect repeatability. The average content of Astragaloside A was 0.216% with RSD 1.5%. The tests were stable at 0, 2, 4, 6, and 8 h.

### Patients

Before the experiment, all procedures were approved by the human research ethical committee of the Affiliated Hospital of Changchun University of Traditional Chinese Medicine (Changchun, China, approval no. 20170128x). A written consent form approved by our committee and signed by all subjects. Eighty children with allergic asthma, were recruited at the Department of Pediatrics, Affiliated Hospital of Changchun University of Traditional Chinese Medicine. The clinical diagnostic criteria of the “Children's Asthma Diagnosis and Prevention Guide” was used to examine allergic asthma in children (22), and the children had the following symptoms: wheezing, shortness of breath, chest pain or cough, and allergic asthma caused by cold air, physical and chemical stimulation, viral upper respiratory tract infection and exercise. Wheezing episodes in the lungs could be heard and the expiratory phase was prolonged. The typical clinical manifestations, such as no clear wheezing or signs should have at least the following positive test: bronchial provocation test or motor positive test, and or bronchial diastolic positive test (forced expiratory volume in 1 s FEV1 increased).

### Allergy skin test

The hypersensitivity response of each patient was assessed by using conventional skin prick tests against 16 common aeroallergens according to an earlier report (23). Skin prick tests were performed according to the methods introduced by Gislason (24). The test would be regarded as clinically significant if the allergen reactions were more than 10.

### Measurement of total IgE

Serum IgE levels were measured by using the Human IgE ELISA Kit from Abcam (Shanghai, China). Serum IgE levels were positively associated with the color intensity of the test and calibrated against the WHO standard for IgE (25).

### The test for allergen-specific IgE

Allergen-specific IgE was detected in the patients' serum against HD, *D. pteronyssinus* (DP), *D. farinae* (DF), and *B. tropicalis* (BT) by using the AllergyScreen system and ImmunoCAP 100 system (Amersham Pharmacia Biotech, Uppsala, Sweden). The test was calibrated against the WHO Standard for IgE ranging from 0.35 to 100 KU/L for specific IgE.

### Inclusion criteria

The following inclusion criteria were used:

(1) Male or female patients between the ages of 3–12 years; (2) Allergens reactions more than 10. (3)Total serum IgE levels more than 350 IU/mL; (4) competency to perform pulmonary function tests and pulmonary function FEV1 < 80% predictive value; (5) children not taking the medicine such as aspirin and other drugs that may trigger asthma symptoms within 4 weeks before enrollment; (6) children able to receive treatment under the guardianship of a parent who signed an informed consent form.

### Exclusion criteria

Children who had the following conditions were excluded: (1) various psychiatric disorders; (2) severe congenital heart disease; (3) pneumothorax, pleural effusion, active pulmonary tuberculosis, acute exacerbation within the past month and received emergency treatment; (5) infectious rhinitis and sinusitis; (6) anaphylactic shock or eczema of unknown etiology; (7) received immunotherapy and (8) adverse reactions after enrollment.

### Patients grouping

The extracts of AM were evaporated to dry, and the residue was dissolved in 10 mL ethanol. AOS was prepared by the 10-fold dilution of AM ethanol solution. Residual petroleum and n-butanol compounds were measured by gas chromatography (Agilent 6890, Agilent Tech., USA) with a flame ionization detecting system. None of the residual compounds were found in the AOS solution.

Based on inclusion and exclusion criteria, 80 children with allergic asthma caused by dust mites were recruited at our hospital from February 2016 to June 2017. The sample size was analyzed by using PASS version 13 (NCSS Statistical Software, Kaysville, UT, USA), and the power of sample size was 85%. The children were evenly and randomly assigned into two groups: the AOS group, where the patients received 10 mL AOS daily and the control group, where the patients received placebo (0.1% quinine chloride in deionized water) daily. The duration of the experiment was 6 months.

### Evaluation of allergic asthma symptoms

Lung function was measured by using FEV1, which was compared with predicted values. Most symptoms of allergic asthma were investigated by using Pediatric Asthma Quality of Life Questionnaire (PAQLQ) scores as shown in Table 1 (26).

**Table 1:** The Pediatric Asthma Quality of life questionnaire (PAQLQ score).

**Scores**	**Aspects**
**LIMITED ACTIVITY**	
1	Strong physical activity
2	Medium physical activity
3	Social activity
4	Work-related activities
5	Sleepiness
**SYMPTOMS**	
8	Shortness of breath
14	Dull aching chest
18	Expiratory or inspiratory difficulties, early morning symptoms of asthma
24	Night arousal
**ENVIRONMENT**	
9	Smoke,
17	Dust
23	Air pollution environment
26	Strong smell symptoms,
25	Air pollution of the environment
28	Strong smell had to be avoided
**EMOTION**	
6	Chest tightness caused by the degree of discomfort
7	Worried about suffering from asthma
12	Cough caused by discomfort
13	Asthma and worry
15	Medication
16	Clear throat
21	Worry about no asthma medication
22	Heavy breathing
27	Fear of breathlessness
29	Remuneration,
30	Desperately breathing,
10	Limitation of conscious activities

The severity of asthma was classified into four levels according to previous report (27): mild intermittent, mild persistent, moderate persistent, and severe persistent based on symptom frequency and either spirometric (FEV_1_) or peak expiratory flow (PEF). The primary outcome was also measured according to asthma-related clinical events, including cough, wheeze, and need of intervention. Allergic sensitization to common dietary and respiratory allergens were measured according to an earlier report (28). Serum IgE of 0.2 or 0.35 IU/mL were regarded as positive and predictive for allergic asthma. Eosinophils (29) and serum levels of ECP (30) are increased in allergic asthma.

### Measurement of serum levels of IL-10 and TGF-β

Five millimeters of venous blood was obtained from each patient. Two millimeters of blood was placed at room temperature for 1 h. The serum was isolated via centrifugation at 5000 × *g* for 10 min. The serum levels of IL-10 and TGF-β were measured by using the ELISA kits from R & D Systems, Inc. (Minneapolis, MN, USA). The serum Th1 cytokines (IL-2 and IFN-γ), and Th2 cytokines (IL-4 and IL-6) (31) were measured by using the kits from Abcam (Shanghai office, China).

### Measurement of the percentage of gated CD4^+^ T cells, CD4^+^cd25^+^ T cells, CD4^+^cd25^high^ treg cells, CD4^+^cd25^+^ foxP3^+^ treg cells and CD4^+^CD25^high^ CD127^low^ treg cells

Three millimeters of blood was added to an anticoagulant-containing (EDTA-K2) tube. Peripheral blood mononuclear cells (PBMCs) were isolated from human peripheral blood via Ficoll-Hypaque (Sigma, St. Louis, MO, USA) density gradient centrifugation. Mouse anti-human CD4 monoclonal antibody was purchased from Zhongshan Golden Bridge Biotechnology (Beijing, China) CD127 (BD Pharmingen #558598, San Jose, CA) was conjugated to ALEXA FLUOR 488. Goat-anti-mouse FITC IgG was purchased from Kangwei (Beijing, China). CD25 PE-Cy7 (BD, clone M-A251), CD25 APC (clone 2A3), and FoxP3 PE (clone PCH101) were purchased from BD Biosciences (Franklin, NJ, USA). CD4+ T cells were gated on side scatter height vs. CD4. The percentage of CD4^+^CD25^+^ T cells, CD4^+^CD25^high^ Treg cells, CD4^+^CD25^+^ FoxP3^+^ Treg cells and CD4^+^CD25^high^CD127^low^ Treg cells in PBMCs was measured by using FACSCalibur flow cytometer (Becton Dickinson, Mountain View, CA, USA).

### Statistical analysis

SPSS20.0 statistical software was used to perform statistical analyses. All data were expressed as mean values ± standard deviation (S.D.). Normal distribution and variance homogeneity were analyzed by a paired *t*-test. Normal distribution was analyzed by ANOVA between groups. Non-normal distribution was analyzed by Wilcoxon rank test. Count data were compared by using contingency χ^2^ test. The statistical differences were significant if *P* < 0.05.

## Results

### Recovery rate of astragaloside A

AOS was taken from six different batches. With two copies per group, each group was added to a concentration of 0.197 mg/mL Astragaloside A control solution 0.8, 1.0, and 1.2 mL. The average recovery rate of Astragaloside A was 96.2% with RSD 2.65% (Table 2).

**Table 2:** Recovery rate of Astragaloside A.

**Times**	**Sampling volume/ml**	**Sample content/mg**	**Addition/mg**	**Measured values/mg**	**Recovery rate**	**Average value**	**RSD/%**
1	10	0.215	0.1574	0.35420	0.9511	0.962	2.65
2	10	0.215	0.1574	0.36540	0.9812		
3	10	0.215	0.1967	0.39870	0.9684		
4	10	0.215	0.1967	0.37540	0.9118		
5	10	0.215	0.2360	0.44670	0.9905		
6	10	0.215	0.2360	0.43650	0.9678		

### Average contents of astragaloside a in AOS

Table 3 showed that the contents of Astragaloside A were 0.216 ± 0.027 mg/mL from six batches of AOS.

**Table 3:** The average contents of Astragaloside A from 6 batches of AOS.

**Batches**	**1**	**2**	**3**	**4**	**5**	**6**
Contents of Astragaloside A, mg/ml	0.203	0.242	0.213	0.189	0.243	0.204

*AOS, astragalus oral solution*.

### Clinical demographic characteristics

The AOS and control groups were composed of 80 cases with 43 males and 37 females, and aged 3–12 years. Age is associated with the risk of allergic asthma (32, 33). In our study, age distribution (5-7, 8-10, and 11-12) was similar between the two groups (*P* > 0.05). The experimental group comprised 23 males and 17 females, with an average age of 8.7 ± 3.3 years. The control group comprised 22 males and 18 females, with an average age of 8.9 ± 2.7 years. The statistical difference was not statistically significant in all parameters (Table 4, *P* > 0.05). The most allergic responses to common inhalants included *Cocos nucifera, Brassica nigra*, cat dander, HD, DP, DF, and BT. The statistical difference for these inhalants was insignificant between two groups (Table 5, *P* > 0.05). The total serum IgE levels were 428.49 ± 58.61 and 431.27± 60.45 IU/mL between two groups. The statistical difference for allergen-specific IgE reactivity against HD, DP, DF, and BT was insignificant between the two groups (Table 6, *P* > 0.05).

**Table 4:** Clinical demographic characteristics.

	**AOS group**	**Control group**	**χ^2^ values or *t* values**	***P*-values**
Age, years	8.7 ± 3.3	8.9 ± 2.7	0.124	0.658
5-7 year, *n* (%)	6	4	0.630	0.730
8-10 year, *n* (%)	26	29		
11-12 year, *n* (%)	8	7		
Male/Female	23/17	22/18	0.051	0.822
BMI	22.67 ± 3.18	23.98 ± 2.75	0.578	0.209
Disease duration, months	36.90 ± 4.28	38.91 ± 5.27	2.397	0.009
Respiratory rate, times/min	19.74 ± 1.54	20.33 ± 1.49	−1.908	0.153
Heart rate, time/min	76.82 ± 3.77	77.02 ± 3.03	−0.292	0.768
Systolic pressure, mmHg	121.68 ± 9.35	123.83 ± 11.55	−1.039	0.326
Diastolic pressure, mmHg	77.04 ± 7.11	75.46 ± 6.51	1.127	0.235

*BMI, body mass index = weight (kg)/height (m^2^)*.

**Table 5:** Allergic response to common inhalants.

	**AOS group**	**Control group**	***P*-values**
Pollens			0.936
Cocos nucifera	31 (77.5)	33 (82.5)	
Brassica nigra	20 (50)	24 (60)	
Delonix sp.	19 (47.5)	17 (42.5)	
*Azadirachta indica*	17 (42.5)	15 (37.5)	
*Caesalpinia* sp	15 (37.5)	18 (45)	
Molds			0.915
*Aspergillus fumigatus*	11 (27.5)	12 (30)	
*Aspergillus niger*	8 (20)	9 (22.5)	
*Candida albicans*	5 (12.5)	4 (10)	
*Cladosporium* sp.	6 (15)	7 (17.5)	
*Alternaria lternate*	4 (10)	2 (5)	
Others			0.623
Dog dander	6 (15)	9 (22.5)	
Cat dander	20 (50)	21 (52.5)	
House dust	40 (100)	32 (80)	
*Dermatophagoides pteronyssinus*	32 (80)	39 (97.5)	
*Dermatophagoides farinae*	21 (52.5)	19 (47.5)	
*Blomia tropicalis*	24 (60)	22 (55)	

*Chi-square test was performed and there will be significant difference if P < 0.05 between two groups*.

**Table 6:** Allergen-specific IgE reactivity against HD, DP, DF, and BT Allergens.

	**AOS group**	**Control group**	***P*-values**
HD	28 (70)	25 (62.5)	0.478
DP	24 (60)	20 (50)	0.369
DF	30 (75)	33 (82.5)	0.412
BT	35 (87.5)	28 (70)	0.056

*HD, House dust; DP, D. pteronyssinus; DF, D. farinae; BT, B. tropicalis. Chi-square test was performed and there will be significant difference if P < 0.05 between two groups*.

No significant differences were found for symptoms of allergic asthma and biochemical characterization of allergic asthma between the two groups (Tables 7, 8). Significant differences of asthma-related events were found after 6-month follow-up (Table 7). The severity of allergic asthma was significantly lower in the experimental group than in the control group (*P* < 0.05).Similarly, biochemical indices of allergic asthma were significantly lower in the experimental group than in the control group (Table 8, *P* < 0.05).

**Table 7:** Comparison of symptoms of allergic asthma between two groups.

	**AOS group, *n* = 40**	**Control group, *n* = 40**	***P*-values**
**BEFORE THERAPY**			
No. of wheezing episodes	1.10 (0.7–1.6)	1.08 (0.6–1.5)	0.85
Days with wheeze	7.58 (1.36–13.9)	6.95 (0.90–12.3)	0.25
Days on inhalative betamimetics	13.8 (3.37–24.6)	13.2 (8.18–17.2)	0.78
Days on inhalative steroids	10.0 (7.23–24.9)	9.80 (6.99–17.5)	0.89
No. of rescue-free days	128.7 (108.4–158.6)	121.5 (112.0–163.0)	0.93
No. of symptom-free days	123.0 (126.4–162.6)	128.5 (115.7–160.3)	0.71
**Allergic Asthma Classification, Cases (%)**			
Mild intermittent	13 (32.5)	15 (37.5)	0.968
Mild persistent	12 (30)	11 (27.5)	
Moderate persistent	8 (20)	7 (17.5)	
Severe persistent	7 (17.5)	7 (17.5)	
**AFTER THERAPY**			
No. of wheezing episodes	0.51 (0.3–1.9)	1.10 (0.6–1.9)	0.03
Days with wheeze	5.26 (3.03–13.7)	11.3 (7.15–14.8)	0.01
Days on inhalative betamimetics	10.7 (6.3–30.2)	13.9 (2.68–18.9)	0.02
Days on inhalative steroids	10.5 (6.3–22.7)	14.8 (4.6–25.6)	0.01
No. of rescue-free days	148.5 (108.9–168.1)	126.0 (99.6–172.4)	0.02
No. of symptom-free days	142.5 (102.6–152.5)	104.0 (86.2–141.8)	
**Allergic Asthma Classification, Cases (%)**			
Mild intermittent	21 (52.5)	12 (30)	0.02
Mild persistent	10 (2.5)	6 (1.5)	
Moderate persistent	4 (10)	14 (3.5)	
Severe persistent	5 (12.5)	8 (20)	

*The statistical difference was significant if P < 0.05*.

**Table 8:** Comparison of biochemical characterization of allergic asthma between two groups.

	**AOS group, *n* = 40**	**Control group, *n* = 40**	***P*-values**
**BEFORE THERAPY**			
Total IgE (IU/mL)	416.7 (312.8–556.4)	412.2 (310.0–560.6)	0.86
Eosinophils (%)	2.6 (2.3–3.9)	2.8 (2.3–3.5)	0.32
ECP (μg/mL)	22.3 (14.9–29.9)	21.9 (12.6–28.2)	0.69
**AFTER THERAPY**			
Total IgE (IU/mL)	214.7 (162.3–290.5)	420.2 (316.2–481.3)	0.01
Eosinophils (%)	2.2 (1.5–4.0)	3.0 (2.5–3.6)	0.01
ECP (μg/mL)	16.7 (9.89–23.8)	24.8 (16.6–32.4)	0.01

*The statistical difference was significant if P < 0.05*.

### AOS treatment improved the allergic asthma symptoms in children

**Figure 1:**
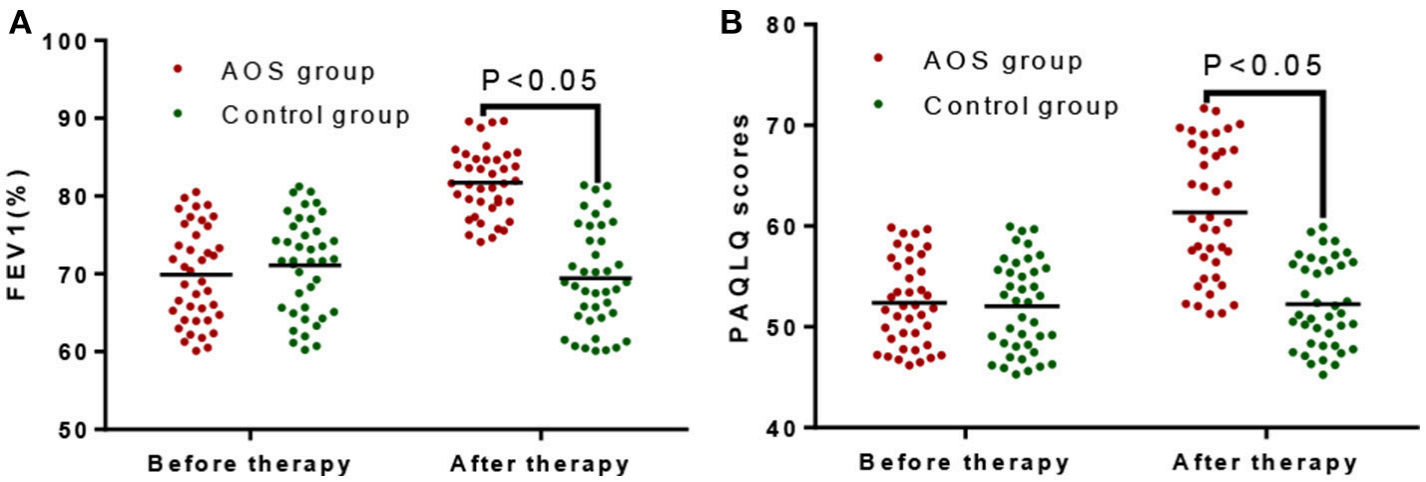
The effects of AOS on FEV1% and PAQLQ scores in the children with allergy asthma. **(A)** The effects of AOS on FEV1% in the children with allergy asthma. **(B)** The effects of AOS on PAQLQ scores in the children with allergy asthma. FEV1, forced expiratory volume in 1 s; PAQLQ, Pediatric Asthma Quality of Life Questionnaire. *n* = 40 for each group. The statistical difference was significant for *P* < 0.05.

Before treatment, the FEV1% in the AOS group was comparable with that in the control group (*P* > 0.05, Figure 1A). After treatment, the FEV1% were significantly increased in the AOS group than in the control group (*P* < 0.05, Figure 1A). Similarly, PAQLQ scores were comparable between two groups before treatment (*P* > 0.05, Figure 1B). After treatment, PAQLQ scores were increased significantly in the AOS group when compared with those in the control group (*P* < 0.05, Figure 1B). The results suggest that AOS reduces the symptoms of allergic asthma by improving FEV1% and PAQLQ.

### AOS increased the serum level of IL-10 and reduced the level of TGF-β

**Figure 2:**
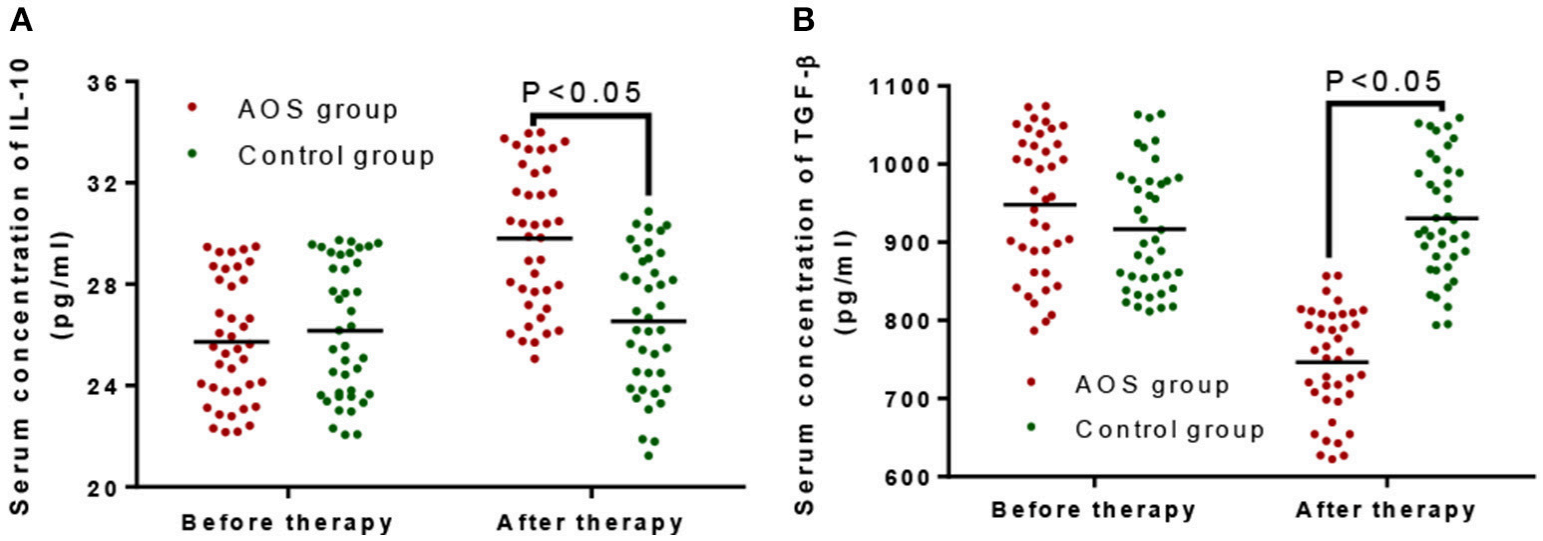
The effects of AOS on the serum levels of IL-10 and TGF-β in the children with allergy asthma. **(A)** The effects of AOS on serum level of IL-10 in the children with allergy asthma. **(B)** The effects of AOS on TGF-β in the children with allergy asthma. *n* = 40 for each group. The statistical difference was significant for *P* < 0.05.

The serum levels of IL-10 were comparable between the AOS (24.12 ± 3.68 pg/mL) and control groups (26.25 ± 3.79 pg/mL) (Figure 2A, *P* > 0.05). Comparatively, the serum levels of TGF-β were same between the AOS (932.67 ± 148.43 pg/mL) and control groups (968.27 ± 150.64 pg/mL) (Figure 2B, *P* > 0.05). After treatment, the serum level of IL-10 in the experimental group was significantly increased and the level of TGF-β was significantly decreased compared with those in the control group (Figure 2, *P* < 0.05). The results suggest that AOS treatment increases the serum levels of IL-10 and reduces the levels of TGF-β.

### AOS treatment reduced serum level of th1 (IL-2 and IFN-γ) and th2 cytokines (IL-4 and IL-6)

**Figure 3:**
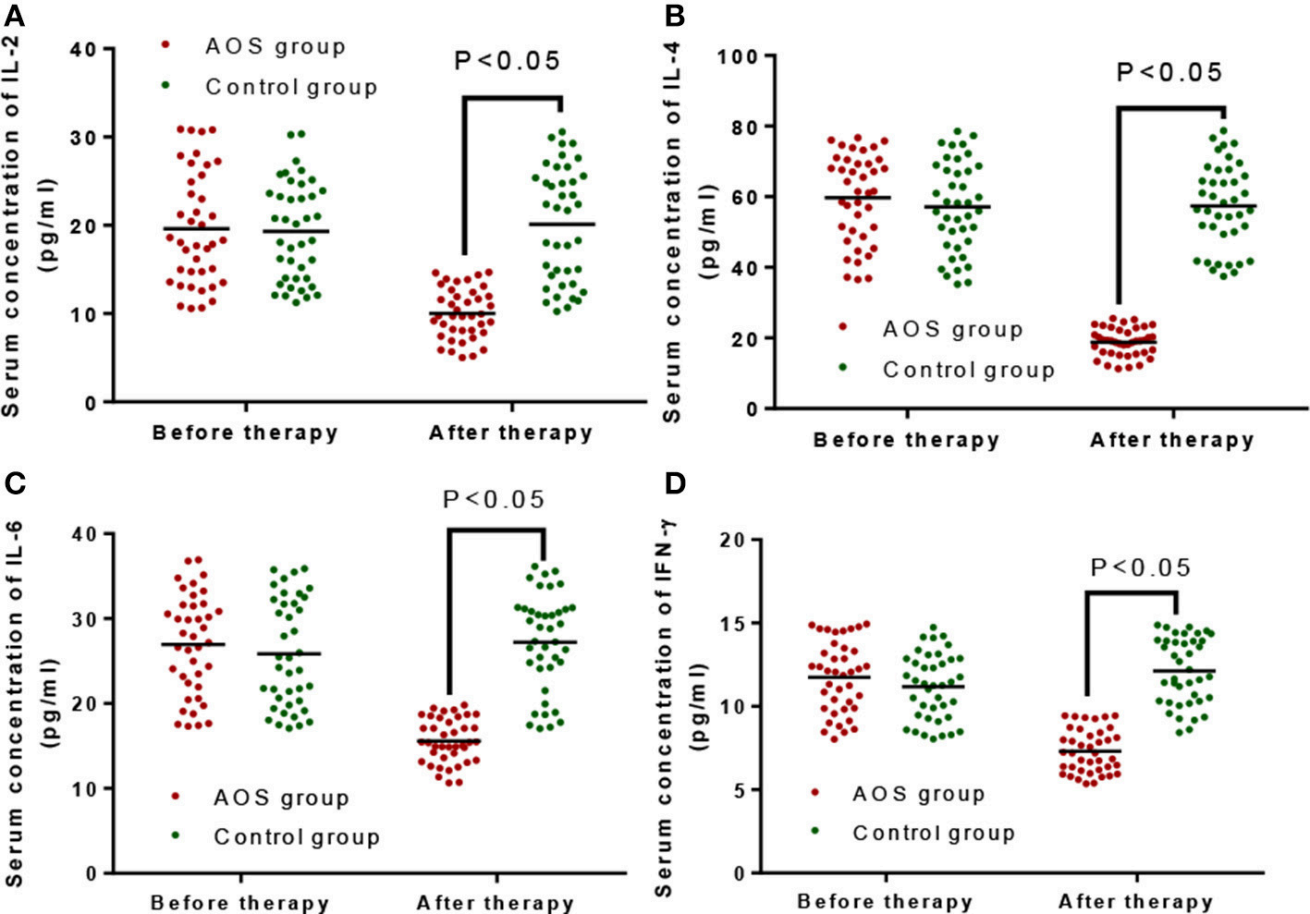
The effects of AOS on the serum levels of Serum Th1 cytokines (IL-2 and IFN-γ), and Th2 cytokines (IL-4 and IL-6). **(A)** The effects of AOS on serum level of IL-2. **(B)** The effects of AOS on serum level of IL-4. **(C)** The effects of AOS on serum level of IL-2. **(B)** The effects of AOS on serum level of IL-6. **(D)** The effects of AOS on serum level of IFN-γ. *n* = 40 for each group. The statistical difference was significant for *P* < 0.05.

Before the treatment, the statistical difference for the serum level of Th1 (IL-2, Figure 3A, and IFN-γ, Figure 3D) and Th2 cytokines (IL-4, Figure 3B and IL-6, Figure 3C) was insignificant between AOS and control groups (*P* > 0.05). After the treatment, the serum levels of Th1 (IL-2, Figure 3A, and IFN-γ, Figure 3D) and Th2 cytokines (IL-4, Figure 3B and IL-6, Figure 3C) were lower in the AOS group than the control group (*P* < 0.05).

### Percentage of gated CD4^+^ T cells, CD4^+^CD25^+^ T cells, CD4^+^CD25^high^, CD4^+^CD25^+^foxP3^+^ treg cells and CD4^+^CD25^high^CD127^low^ treg cells

**Figure 4:**
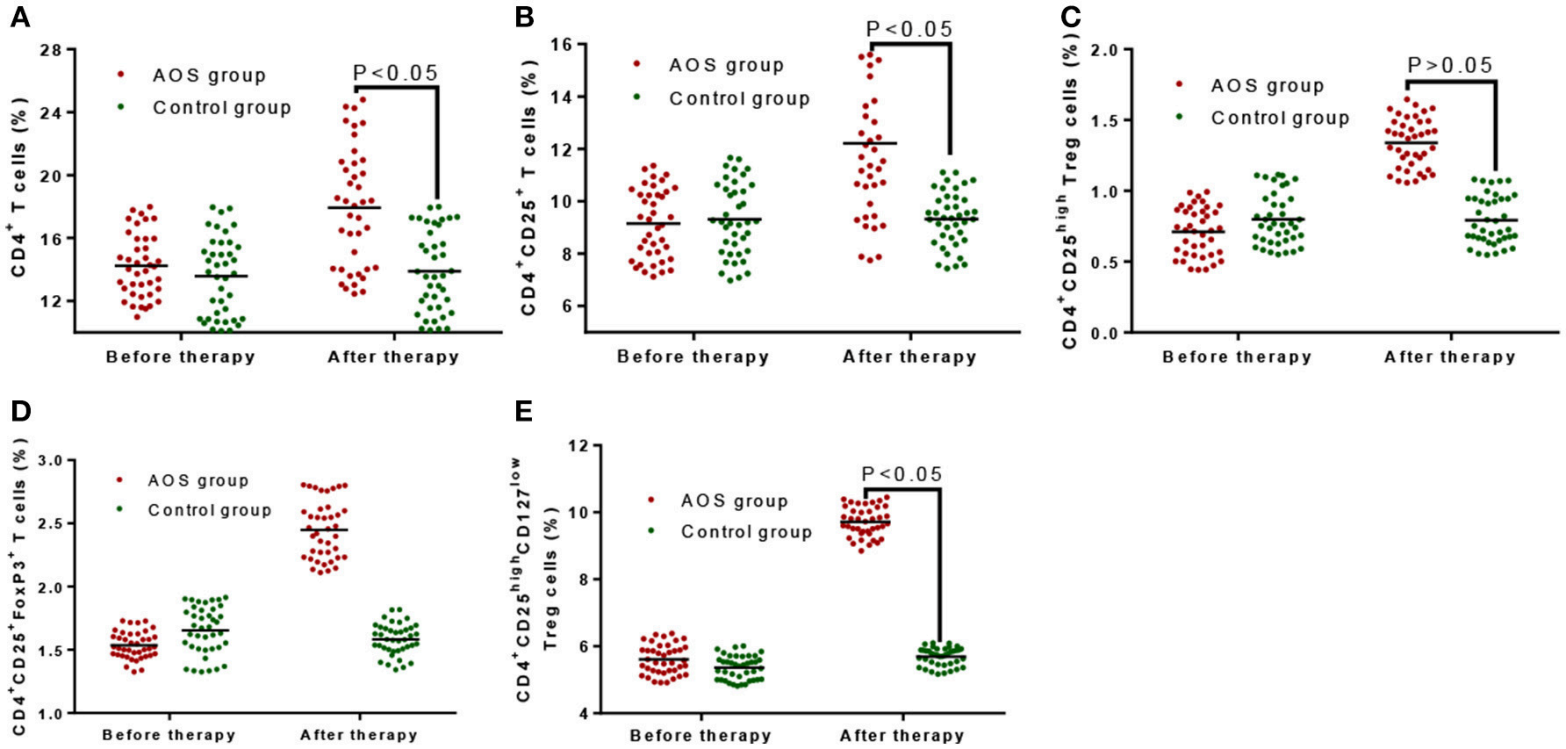
The effects of AOS on the contents of T cells in the children with allergy asthma. **(A)** The effects of AOS on the contents of gated CD4^+^ T cells in the children with allergy asthma. **(B)** The effects of AOS on the contents of CD4^+^CD25^+^ T cells in the children with allergy asthma. **(C)** The effects of AOS on the contents of CD4^+^CD25^high^ Treg cells in the children with allergy asthma. **(D)** The effects of AOS on the contents of CD4^+^CD25^+^FoxP3 Treg cells in the children with allergy asthma. **(E)** The effects of AOS on the contents of CD4^+^CD25^high^CD127^low^ Treg cells in the children with allergy asthma. *n* = 40 for each group. The statistical difference was significant for *P* < 0.05.

Before treatment, the percentage of gated CD4^+^ T cells (14.26 ± 3.51%, Figure 4A), CD4^+^CD25^+^ T cells (9.15 ± 2.28%, Figure 4B), CD4^+^CD25^high^ (5.67 ± 0.75%, Figure 4C), CD4^+^CD25^+^FoxP3^+^ Treg cells (1.53 ± 0.21%, Figure 4D), and CD4^+^CD25^high^CD127^low^ (0.72 ± 0.28%, Figure 4E) Treg cells in the AOS group were comparable with those in the control group, gated CD4^+^ T cells (13.89 ± 4.01%, Figure 4A), CD4^+^CD25^+^ T cells (9.29 ± 2.37%, Figure 4B), CD4^+^CD25^high^ (5.38 ± 0.64%, Figure 4C), CD4^+^CD25^+^FoxP3^+^ Treg cells (1.62 ± 0.30%, Figure 4D), and CD4^+^CD25^high^CD127^low^ (0.79 ± 0.25%, Figure 4E) (*P* > 0.05). After treatment, the percentage of gated CD4^+^ T cells (18.63 ± 6.27%, Figure 4A), CD4^+^CD25^+^ T cells (12.34 ± 4.61%, Figure 4B), CD4^+^CD25^high^ (9.65 ± 0.83%, Figure 4C), CD4^+^CD25^+^FoxP3^+^ Treg cells (2.46 ± 0.37%, Figure 4D) and CD4^+^CD25^high^CD127^low^ (1.34 ± 0.31%, Figure 4E) Treg cells in the AOS group were significantly higher than those in the control group, gated CD4^+^ T cells (13.99 ± 4.22%, Figure 4A), CD4^+^CD25^+^ T cells (9.12 ± 2.05%, Figure 4B), CD4^+^CD25^high^ (5.63 ± 0.47%, Figure 4C), CD4^+^CD25^+^FoxP3^+^ Treg cells (1.58 ± 0.24%, Figure 4D), and CD4^+^CD25^high^CD127^low^ (0.81 ± 0.28%, Figure 4E) (*P* < 0.05) (*P* < 0.05). The results suggest that AOS consumption increases the percentage of gated CD4^+^ T cells, CD4^+^CD25^+^ T cells, CD4^+^CD25^high^, CD4^+^CD25^+^FoxP3^+^ Treg cells, and CD4^+^CD25^high^CD127^low^ Treg cells in children with allergic asthma.

## Discussion

The results showed that the content of Astragaloside A in AOS varied from 0.19 to 0.24 mg/mL. The quality of the different batches of the mixture was influenced by the quality of the decoction pieces, processing and other factors. HPLC was an effective method to detect the quality of AOS and provided a basis for high-quality standards.

Allergic asthma is caused by a variety of cells, including inflammatory cells (neutrophils, T lymphocytes, eosinophils, and mast cells) (34), airway structural cells (airway smooth muscle cells and epithelial cells) (35), and cell components. AR is the most common symptoms of allergic asthma after individuals come in contact with allergens. Many inflammatory cells and cytokinesare involved in the inflammatory responses, including nasal itching, sneezing, running nose, and stuffy nose. The incidence of childhood allergic asthma has been increasing, but its pathogenesis is not yet clear. The traditional Th1/Th2 imbalance theory cannot explain all the pathogenesis. Presently, a number of studies have focused on Treg cells to investigate the pathognesis (36, 37). Our findings demonstrated that AOS treatment reduced the serum levels of Th1 and Th2 cytokines (Figure 3). Analyses of childhood allergic asthma by using CD4^+^CD25^high^CD127^low^ Treg cells will have important clinical values.

IL-10 regulates allergic asthma and is directly involved in the regulation of inflammatory cells. IL-10 factor can inhibit the proliferation, secretion of Th cells, and the proliferation and differentiation of antigen-presenting cells (38). IL-10 cells inhibit T cells, antigen presentation (39), and the expression of IL-8 (40). IL-10 was reported to inhibit eosinophil-induced inflammatory effects of asthma, suggesting that IL-10 plays a key negative regulatory role in the development of allergic asthma (41). The level of IL-10 was found to be reduced significantly in T cells of peripheral blood during the onset of allergic asthma (42). In our study, the IL-10 level in the AOS group was comparable with that in control group before treatment (*P* > 0.05, Figure 2). After treatment, the level of IL-10 in the AOS group was significantly increased (*P* < 0.05). After AOS treatment, IL-10 may inhibit the antigen-presenting process and T cells response and play a critical role in the prevention of asthma.

TGF-β is a kind of stimulating factor with many biological effects, and it plays a critical role in regulating inflammation in various organs and tissues. Thus, TGF-β is widely studied in the transforming growth factor family and involved in the fibrosis formation of tissues and organs. Its overexpression in cardiac tissue can cause cardiac hypertrophy or myocardial fibrosis, which is involved in the process of ventricular remodeling (43, 44). TGF-β is a multifunctional cytokine that can participate in the process of cell proliferation, apoptosis and differentiation (45). In the immune system, TGF-β is a regulator of immunity with both pro- and anti-inflammatory effects and is involved in a variety of airway inflammation and immune responses. Many studies on TGF-β in the pathogenesis of asthma have been performed. However, t many controversies at different stages of development are still present. Most of researchers think that TGF-β can inhibit or promote inflammation (46, 47).

Elevated levels of TGF-β were reported in patients with atopic asthma (48, 49), which may be caused by the continued stimulation of allergens and airway remodeling. TGF-β also has a chemotactic function and can promote the proliferation and differentiation of inflammatory cytokines (50). In previous studies, the severity of asthma was found to be related to the concentration of TGF-β in the peripheral blood (51). In the present study, the serum level of TGF-β in the experimental group was comparable with that in the control group (*P* > 0.05). After AOS consumption, the level of TGF-β was significantly decreased (*P* < 0.05), suggesting that AOS ameliorates allergic asthma by reducing the serum level of TGF-β.

Treg cells are a subset of T-cells that control the autoimmune reactivity *in vivo* and play a key role in the inhibition of autoimmunity (52, 53). They exert immunosuppressive effects through the specific binding of their surface molecules (CTLA-4, CD25) to the corresponding ligands on the cells (54). IL-10 can inhibit the proliferation of T cells, the synthesis of cytokines such as IL-2 by Th1 and Th2 cells, and the expression of MHCII in macrophages (55). Immune regulatory T cells may maintain the immune tolerance via several mechanisms. CD4^+^CD25^+^ Treg cells are able to bind to target cells and participate in immune regulation. In previous studies, CD4^+^CD25^+^ Treg cells and CD4^+^CD25^+^ T cells were increased simultaneously with CD4^+^CD25^+^ Treg proliferation (56). The percentage of CD4^+^CD25^+^ T cells and CD4^+^CD25^high^CD127^low^ was decreased during acute episode of childhood allergic asthma (57). In the present study, the percentage was comparable for CD4^+^CD25^+^ T cells, CD4^+^CD25^high^, CD4^+^CD25^+^FoxP3^+^ Treg cells and CD4^+^CD25^high^CD127^low^ Treg cells between two groups before the treatment (Figure 4, *P* > 0.05). After the treatment, AOS consumption increased the percentage of CD4^+^CD25^+^ T cells, CD4^+^CD25^high^, CD4^+^CD25^+^FoxP3^+^ Treg cells and CD4^+^CD25^high^CD127^low^ Treg cells (Figure 4, *P* < 0.05). The results suggest AOS consumption reduces the risk of allergic asthma by increasing the percentage of CD4^+^CD25^+^ T cells, CD4^+^CD25^high^, CD4^+^CD25^+^FoxP3^+^ Treg cells and CD4^+^CD25^high^CD127^low^ Treg cells in PBMCs.

Th2 cytokines are associated with allergic airway inflammation (2) while Th1 cells inhibit Th2 immune activities (3). The imbalance of Th1/Th2 cytokines indicates the risk of asthma (58). Peripheral blood eosinophils increase the levels of Th1 and Th2 cytokines (4). By contrast, AOS therapy will reduce the levels of peripheral blood eosinophils and may result in the down-regulation of Th1 and Th2 cytokines. AOS may also increase the level of Treg cells, which will suppress the level of blood eosinophils (5).

The present study has some limitations to. For the side effects of AOS were still unclear. According to previous reports, *Astragalus* induces some side effects, including anemia, neutropenia, thrombocytopenia, fatigue, poor appetite, nausea, and vomiting (1). However, these side effects were not found in the present study. Although Astragaloside A is the main bioactive component in AOS, other components were not analyzed in AOS. The functions of certain components should be confirmed in the future work. In this experiment, we only explored the effects of AOS on the serum levels of IL-10, TGF-β and the percentage of CD4^+^CD25^+^ T cells, CD4^+^CD25^high^, CD4^+^CD25^+^FoxP3^+^ Treg cells and CD4^+^CD25^high^CD127^low^ Treg cells in PBMCs. However, modern studies show that the expression level of Treg cells is associated with a variety of autoimmune diseases. The study of Treg alone cannot fully explain the exact mechanism for its action. In recent years, the research on the immune balance between Treg/Th17 has been increased. In the future, further work is highly demanded to reveal the relationship between the impact of the balance and allergic asthma in children.

## Conclusions

The average contents of Astragaloside A were 0.216 ± 0.027 mg/mL from six batches of AOS. After 6-month therapy, AOS treatment was more effective in the experimental group than in the control group (*P* < 0.05). AOS reduced the symptoms of allergy asthma in children group by improving FEV1% and PAQLQ scores. The children with allergic asthma have a lower level of serum IL-10 and higher level of TGF-β. AOS consumption increased the level of serum IL-10 and reduced the level of TGF-β. AOS can be effective in the treatment of allergic asthma in children by increasing the percentage of CD4^+^CD25^+^ T cells, CD4^+^CD25^high^, CD4^+^CD25^+^FoxP3^+^ Treg cells, and CD4^+^CD25^high^CD127^low^ Treg cells.

## Author contributions

WW conceived, designed the study, and wrote the manuscript. QL and WJ performed the experiments, analyzed the data, and contributed reagents, materials, analysis tools.

### Conflict of interest statement

The authors declare that the research was conducted in the absence of any commercial or financial relationships that could be construed as a potential conflict of interest.

## References

[1] Lin YD Fan XL Zhang H Fang SB Li CL Deng MX. The genes involved in asthma with the treatment of human embryonic stem cell-derived mesenchymal stem cells. Mol Immunol. (2018) 95:47–55. 10.1016/j.molimm.2018.01.013 29407576

[2] Normansell R Kew KM Bridgman AL Sublingual immunotherapy for asthma. Cochrane Database Syst Rev. (2015) 8:CD011293 10.1002/14651858.CD011293.pub2 PMC6769158 26315994

[3] Powell C Milan SJ Dwan K Bax L Walters N Mepolizumab versus placebo for asthma. Cochrane Database Syst Rev. (2015) 7:CD010834 10.1002/14651858.CD010834.pub2 26214266

[4] Marple BF. Allergic rhinitis and inflammatory airway disease: interactions within the unified airspace. Am J Rhinol Allergy (2010) 24:249–54. 10.2500/ajra.2010.24.3499 20819460

[5] Bousquet J Van Cauwenberge P Khaltaev N World Heath Organization. Allergic rhinitis and its impact on asthma. J Allergy Clin Immunol. (2001) 108:S147–334. 10.1067/mai.2001.118891 11707753

[6] Rahman MA Chakraborty R Ferdousi KR Alam A Chowdhury MK Paul BK. New therapeutic approach to treat allergic rhinitis & bronchial asthma, considering these two as one united airway disease. Mymensingh Med J. (2017) 26:216–21. 28260781

[7] Calus L Devuyst L Van Zele T De Ruyck N Derycke L Bachert C. The response to nasal allergen provocation with grass pollen is reduced in patients with chronic rhinosinusitis with nasal polyposis and grass sensitization. Clin Exp Allergy (2016) 46:555–63. 10.1111/cea.12687 26661927

[8] Svendsen ER Gonzales M Commodore A. The role of the indoor environment: residential determinants of allergy, asthma and pulmonary function in children from a US-Mexico border community. Sci Total Environ. (2018) 616:1513–23. 10.1016/j.scitotenv.2017.10.162 29107378

[9] Scanlon ST McKenzie AN. Type 2 innate lymphoid cells: new players in asthma and allergy. Curr Opin Immunol. (2012) 24:707–12. 10.1016/j.coi.2012.08.009 22985480

[10] Amin K. Allergic respiratory inflammation and remodeling. Turkish Thor J. (2015) 16:133–40. 10.5152/ttd.2015.4942 29404091 PMC5793767

[11] Hui C McNagny K Denburg J Siracusa M *In situ* hematopoiesis: a regulator of T H 2 cytokine-mediated immunity and inflammation at mucosal surfaces. Mucosal Immunol. (2015) 8:701 10.1038/mi.2015.17 25783967

[12] MacDonald SM. History of histamine-releasing factor (HRF)/translationally controlled tumor protein (TCTP) including a potential therapeutic target in asthma and allergy. Results Probl Cell Differ. (2017) 64:291–308. 10.1007/978-3-319-67591-6_16 29149416

[13] Coquet JM Schuijs MJ Smyth MJ Deswarte K Beyaert R Braun H. Interleukin-21-producing CD4+ T cells promote type 2 immunity to house dust mites. Immunity (2015) 43:318–30. 10.1016/j.immuni.2015.07.015 26287681

[14] Kawayama T Kinoshita T Matsunaga K Naito Y Sasaki J Tominaga Y. Role of regulatory T cells in airway inflammation in asthma. Kurume Med J. (2018) 64:45–55. 10.2739/kurumemedj.MS6430001 29553094

[15] Shu Y Hu Q Long H Chang C Lu Q Xiao R. Epigenetic variability of CD4+CD25+ tregs contributes to the pathogenesis of autoimmune diseases. Clin Rev Allergy Immunol. (2017) 52:260–72. 10.1007/s12016-016-8590-3 27687891

[16] Tian Y Liang X Wu Y. The alternation of autophagy/apoptosis in CD4+CD25+Foxp3+ Tregs on the developmental stages of atherosclerosis. Biomed Pharmacother. (2018) 97:1053–60. 10.1016/j.biopha.2017.11.013 29136784

[17] Agarwal A Singh M Chatterjee BP Chauhan A Chakraborti A. Interplay of T helper 17 cells with CD4(+)CD25(high) FOXP3(+) Tregs in regulation of allergic asthma in pediatric patients. Int J Pediatr. (2014) 2014:636238. 10.1155/2014/636238 24995020 PMC4065696

[18] Zhuge Z Dong Y Li L Jin T. Effects of astragalus polysaccharide on the adhesion-related immune response of endothelial cells stimulated with CSFV *in vitro*. Peer J. (2017) 5:e3862. 10.7717/peerj.3862 29018607 PMC5633024

[19] Lin SM Jiang Y Chen YJ Luo L Doolgindachbaporn S Yuangsoi B. Effects of Astragalus polysaccharides (APS) and chitooligosaccharides (COS) on growth, immune response and disease resistance of juvenile largemouth bass, Micropterus salmoides. Fish Shellfish Immunol. (2017) 70:40–47. 10.1016/j.fsi.2017.08.035 28863890

[20] Jin H Luo Q Zheng Y Nurahmat M Wu J Li B. CD4+ CD25+ Foxp3+ T cells contribute to the antiasthmatic effects of Astragalus membranaceus extract in a rat model of asthma. Int Immunopharmacol. (2013) 15:42–49. 10.1016/j.intimp.2012.11.009 23186751

[21] Lin Y Wang B Luo X. Clinical study of astragalus's preventing the recurrence of asthma in children. Zhongguo Zhong xi yi jie he za zhi Zhongguo Zhongxiyi jiehe zazhi (2011) 31:1090–92. 21910341

[22] Larenas Linnemann DES Del Rio Navarro BE Luna Pech JA Romero Lombard J Villaverde Rosas J Cano Salas MC Recommendations for the prevention and diagnosis of asthma in children: evidence from international guidelines adapted for Mexico. Allergol Immunopathol. (2017) 46:291–303. 10.1016/j.aller.2017.05.011 29288048

[23] Podder S Gupta SK Saha GK. Incrimination of *Blomia tropicalis* as a potent allergen in house dust and its role in allergic asthma in Kolkata Metropolis, India. World Allergy Organization J. (2010) 3:182. 10.1097/WOX.0b013e3181df4d4f 23268430 PMC3488897

[24] Gislason D Gislason T. IgE-mediated allergy to Lepidoglyphus destructor in an urban population–an epidemiologic study. Allergy (1999) 54:878–83. 10.1034/j.1398-9995.1999.00996.x 10485393

[25] Platts-Mills TA de Weck AL Aalberse R Bessot J Bjorksten B Bischoff E Dust mite allergens and asthma—a worldwide problem. J Allergy Clin Immunol. (1989) 83:416–27. 10.1016/0091-6749(89)90128-0 2645343

[26] Sousa KH West SG Moser SE Harris JA Cook SW. Establishing measurement invariance: english and spanish paediatric asthma quality of life questionnaire. Nurs Res. (2012) 61:171–80. 10.1097/NNR.0b013e3182544750 22551991 PMC3361901

[27] Stout JW Visness CM Enright P Lamm C Shapiro G Gan VN. Classification of asthma severity in children: the contribution of pulmonary function testing. Arch Pediatr Adolesc Med. (2006) 160:844–50. 10.1001/archpedi.160.8.844 16894085

[28] Rose MA Stieglitz F Koksal A Schubert R Schulze J Zielen S. Efficacy of probiotic Lactobacillus GG on allergic sensitization and asthma in infants at risk. Clin Exp Allergy (2010) 40:1398–405. 10.1111/j.1365-2222.2010.03560.x 20604800

[29] Tang W Smith SG Du W Gugilla A Du J Oliveria JP. Interleukin-25 and eosinophils progenitor cell mobilization in allergic asthma. Clin Transl Allergy (2018) 8:5. 10.1186/s13601-018-0190-2 29456832 PMC5809891

[30] Sin A Terzioglu E Kokuludag A Sebik F Kabakci T. Serum eosinophil cationic protein (ECP) levels in patients with seasonal allergic rhinitis and allergic asthma. Allergy Asthma Proc. (1998) 19:69–73. 10.2500/108854188778607228 9578914

[31] Yan Y Wang J Qu B Zhang Y Wei Y Liu H. CXCL13 and TH1/Th2 cytokines in the serum and cerebrospinal fluid of neurosyphilis patients. Medicine (2017) 96:e8850. 10.1097/MD.0000000000008850 29381995 PMC5708994

[32] Henckel E Svenson U Nordlund B Berggren Brostrom E Hedlin G Degerman S. Telomere length was similar in school-age children with bronchopulmonary dysplasia and allergic asthma. Acta Paediatr. (2018) 107:1395–401. 10.1111/apa.14294 29476624

[33] Wang P Wu L Ju Y Fu M Shuang T Qian Z. Age-Dependent allergic asthma development and cystathionine gamma-lyase deficiency. Antioxid Redox Signal (2017) 27:931–44. 10.1089/ars.2016.6875 28253731

[34] Tschopp CM Spiegl N Didichenko S Lutmann W Julius P Virchow JC. Granzyme B, a novel mediator of allergic inflammation: its induction and release in blood basophils and human asthma. Blood (2006) 108:2290–9. 10.1182/blood-2006-03-010348 16794249

[35] Lv J Xiong Y Li W Cui X Cheng X Leng Q. IL-37 inhibits IL-4/IL-13-induced CCL11 production and lung eosinophilia in murine allergic asthma. Allergy (2018) 73:1642–52. 10.1111/all.13395 29319845

[36] Dugger KJ Chrisman T Sayner SL Chastain P Watson K Estes R. Beta-2 adrenergic receptors increase TREG cell suppression in an OVA-induced allergic asthma mouse model when mice are moderate aerobically exercised. BMC Immunol. (2018) 19:9. 10.1186/s12865-018-0244-1 29452585 PMC5816563

[37] Datta A Moitra S Das PK Mondal S Omar Faruk SM Hazra I. Allergen immunotherapy modulates sensitivity of Treg cells to apoptosis in a rat model of allergic asthma. Immunotherapy (2017) 9:1239–51. 10.2217/imt-2017-0038 29130799

[38] Wu HY Maron R Tukpah A-M Weiner HL. Mucosal anti-CD3 monoclonal antibody attenuates collagen-induced arthritis that is associated with induction of LAP+ regulatory T cells and is enhanced by administration of an emulsome-based Th2-skewing adjuvant. J Immunol. (2010) 185:3401–07. 10.4049/jimmunol.1000836 20720210 PMC2962584

[39] Fiorentino DF Zlotnik A Vieira P Mosmann TR Howard M Moore KW O'Garra A. IL-10 acts on the antigen-presenting cell to inhibit cytokine production by Th1 cells. J Immunol. (1991) 146:3444–51. 1827484

[40] Wang P Wu P Anthes JC Siegel MI Egan RW Billah MM. Interleukin-10 inhibits interleukin-8 production in human neutrophils. Blood (1994) 83:2678–83. 8167346

[41] Ogawa Y Duru EA Ameredes BT. Role of IL-10 in the resolution of airway inflammation. Curr Mol Med. (2008) 8:437–45. 10.2174/156652408785160907 18691071 PMC9159958

[42] Wang Y-H Voo KS Liu B Chen C-Y Uygungil B Spoede W A novel subset of CD4+ TH2 memory/effector cells that produce inflammatory IL-17 cytokine and promote the exacerbation of chronic allergic asthma. J Exp Med. (2010) 207:2479–91. 10.1084/jem.20101376 20921287 PMC2964570

[43] Rathinavel A Sankar J Mohammed Sadullah SS Niranjali Devaraj S. Oligomeric proanthocyanidins protect myocardium by mitigating left ventricular remodeling in isoproterenol-induced postmyocardial infarction. Fundam Clin Pharmacol. (2018) 32:51–59. 10.1111/fcp.12325 29059499

[44] Sheu JJ Ali HEE Cheng BC Chiang HJ Sung PH Chen KH. Extracorporeal shock wave treatment attenuated left ventricular dysfunction and remodeling in mini-pig with cardiorenal syndrome. Oncotarget (2017) 8:54747–63. 10.18632/oncotarget.18287 28903379 PMC5589618

[45] Blomhoff R. Retinoids may increase fibrotic potential of TGF-beta: crosstalk between two multi-functional effectors. Hepatology (1997) 26:1067–8. 10.1053/jhep.1997.v26.ajhep0261067 9328335

[46] Wang W Koka V Lan HY. Transforming growth factor-β and Smad signalling in kidney diseases. Nephrology (2005) 10:48–56. 10.1111/j.1440-1797.2005.00334.x 15705182

[47] Bira Y Tani K Nishioka Y Miyata J Sato K Hayashi A Nakaya Y Sone S. Transforming growth factor β stimulates rheumatoid synovial fibroblasts via the type II receptor. Modern Rheumatol. (2005) 15:108–13. 10.3109/s10165-004-0378-2 17029045

[48] Manuyakorn W Kamchaisatian W Atamasirikul K Sasisakulporn C Direkwattanachai C Benjaponpitak S. Serum TGF-beta1 in atopic asthma. Asian Pac J Allergy Immunol. (2008) 26:185–9. 19317336

[49] Datau EA Mewengkang H Matheos JC Purnawan I Wibisono M Wongdjaja K. Clinical efficacy and laboratory improvement of bacillus calmette-guerin vaccination on adult atopic asthma: a cohort study. World Allergy Organ J. (2008) 1:63–9. 10.1097/WOX.0b013e31816c8b85 23283393 PMC3650947

[50] Li Y Liu Y Fu Y Wei T Le Guyader L. The triggering of apoptosis in macrophages by pristine graphene through the MAPK and TGF-beta signaling pathways. Biomaterials (2012) 33:402–11. 10.1016/j.biomaterials.2011.09.091 22019121

[51] Hong JG Dong WF Zhou XJ. Effect of montelukast sodium on TGF-beta(1) of peripheral blood mononuclear cells from children with mild persistent asthma. Zhonghua Er Ke Za Zhi (2011) 49:679–84. 22176903

[52] Sprouse ML Scavuzzo MA Blum S Shevchenko I Lee T Makedonas G. High self-reactivity drives T-bet and potentiates Treg function in tissue-specific autoimmunity. JCI Insight (2018) 3:97322. 10.1172/jci.insight.97322. 29367462 PMC5821181

[53] Dash B Shapiro MJ Chung JY Romero Arocha S Shapiro VS. Treg-specific deletion of NKAP results in severe, systemic autoimmunity due to peripheral loss of Tregs. J Autoimmun. (2018) 89:139–48. 10.1016/j.jaut.2017.12.013 29366602 PMC6205721

[54] von Boehmer H Mechanisms of suppression by suppressor T cells. Nat Immunol. (2005) 6:338–44. 10.1038/ni1180 15785759

[55] Taylor A Verhagen J Blaser K Akdis M Akdis CA. Mechanisms of immune suppression by interleukin-10 and transforming growth factor-beta: the role of T regulatory cells. Immunology (2006) 117:433–42. 10.1111/j.1365-2567.2006.02321.x 16556256 PMC1782242

[56] Sasaki Y Sakai M Miyazaki S Higuma S Shiozaki A Saito S. Decidual and peripheral blood CD4+ CD25+ regulatory T cells in early pregnancy subjects and spontaneous abortion cases. MHR (2004) 10:347–53. 10.1093/molehr/gah044 14997000

[57] Huarong H Tiantian L Xiandi M The changes of CD4~+ CD25~+ regulatory T cells in peripheral blood and their significance in the disease severity of asthmatic children. New Med. (2009) 4:005 10.1111/j.1365-2249.2007.03329.x

[58] Ramirez-Velazquez C Castillo EC Guido-Bayardo L Ortiz-Navarrete V. IL-17-producing peripheral blood CD177+ neutrophils increase in allergic asthmatic subjects. Allergy Asthma Clin Immunol. (2013) 9:23. 10.1186/1710-1492-9-23 23822853 PMC3704811

